# Hybrid Speciation in a Marine Mammal: The Clymene Dolphin (*Stenella clymene*)

**DOI:** 10.1371/journal.pone.0083645

**Published:** 2014-01-08

**Authors:** Ana R. Amaral, Gretchen Lovewell, Maria M. Coelho, George Amato, Howard C. Rosenbaum

**Affiliations:** 1 Centro de Biologia Ambiental, Faculdade de Ciências, Universidade de Lisboa, Lisbon, Portugal; 2 Sackler Institute for Comparative Genomics, American Museum of Natural History, New York, New York, United States of America; 3 Mote Marine Laboratory, Sarasota, Florida, United States of America; 4 Ocean Giants Program, Wildlife Conservation Society, New York, United States of America; University of Massachusetts, United States of America

## Abstract

Natural hybridization may result in the exchange of genetic material between divergent lineages and even the formation of new taxa. Many of the Neo-Darwinian architects argued that, particularly for animal clades, natural hybridization was maladaptive. Recent evidence, however, has falsified this hypothesis, instead indicating that this process may lead to increased biodiversity through the formation of new species. Although such cases of hybrid speciation have been described in plants, fish and insects, they are considered exceptionally rare in mammals. Here we present evidence for a marine mammal, *Stenella clymene*, arising through natural hybridization. We found phylogenetic discordance between mitochondrial and nuclear markers, which, coupled with a pattern of transgressive segregation seen in the morphometric variation of some characters, support a case of hybrid speciation. *S. clymene* is currently genetically differentiated from its putative parental species, *Stenella coerueloalba* and *Stenella longisrostris*, although low levels of introgressive hybridization may be occurring. Although non-reticulate forms of evolution, such as incomplete lineage sorting, could explain our genetic results, we consider that the genetic and morphological evidence taken together argue more convincingly towards a case of hybrid speciation. We anticipate that our study will bring attention to this important aspect of reticulate evolution in non-model mammal species. The study of speciation through hybridization is an excellent opportunity to understand the mechanisms leading to speciation in the context of gene flow.

## Introduction

The consequences of the exchange of genetic material between individuals belonging to different species have long been a matter of debate among biologists [1], [2]. Natural hybridization among plant species has been seen as a potential mechanism for the spread of beneficial mutations and the formation of new taxa. In contrast, hybridization in animals has traditionally been viewed as a rare and evolutionarily destructive process leading to the prevention or reversal of divergence between incipient species (e.g. [3]). In recent years, however, hybridization has been well documented across several animal groups, such as insects, birds and fishes, suggesting that this phenomenon may be more important for the evolutionary history and speciation of animals than previously thought [4]. In some cases, hybridization has been shown to increase the genetic diversity of populations [5], providing them with a higher adaptability to environmental change. In other cases, it can lead to the formation of new species, able to explore new niches [6]. Hybridization leading to speciation is a phenomenon that has attracted the attention of scientists for some time now, although it is still poorly understood [7], [8]. It has been reported in plants, fishes, insects and birds, but very rarely in mammals and never in marine mammals [9], [10]. Some argue that the unique genetic (e.g. high rates of gene rearrangements that can lead to a rapid change in gene expression and post-zygotic isolation mechanisms) and morphological mechanisms mammals have, can reduce the potential for the production of viable hybrids [11]. Others argue that hybridization in mammals can be as frequent as in other animals, although its detection can be hindered by cryptic morphological characteristics and by a general lack of extensive studies [4]. Because morphological variation may not always have a genetic basis, hybrid individuals can have the exact same morphotype as one of the parental species [4], thus leading to instances in which hybrids are cryptic. Their identification is thus sometimes only possible with the use of molecular tools.

Cetaceans are a group of marine mammals that have diverged from their terrestrial ancestors around 53 Mya [12]. Their evolution has been characterized by some rapid radiation events, which in some groups has led to a confusing taxonomy and a difficulty in clarifying phylogenetic relationships due to the confounding effects of incomplete lineage sorting and possibly hybridization [13]. Hybridization in cetaceans has been reported to occur both in captivity and in the wild [14], but the extent to which it has contributed to the evolutionary history of these species remains unexplored. Because cetaceans are known to exhibit prominent karyological uniformity [15], they may have the potential to produce viable hybrid offspring more easily than other mammals. This has in fact been confirmed in captivity for at least a cross between *Tursiops truncatus* and *Delphinus capensis*, which produced a fertile hybrid [16]. In the wild there have been several documented cases of hybridization, across whale and porpoise species, but also among dolphin species, including within the genus *Stenella* [17]. While hybridization in whales and porpoises has been confirmed using molecular tools [18], [19], such confirmation is lacking for hybridization among dolphin species.

The clymene dolphin, *Stenella clymene*, Gray (1846) is endemic to the tropical and subtropical Atlantic Ocean. Its cranial features closely resemble those of *Stenella coeruleoalba*, but its external appearance and behaviour are more similar to those of *Stenella longirostris* [20], which has led to some confusion regarding its recognition as a full species. Early molecular studies showed an additional uncertainty in the phylogenetic position of *S. clymene*, with mitochondrial DNA phylogenetic trees placing it as sister taxa of *S. coeruleoalba* [21], [22], and a nuclear DNA tree based on Amplified Fragment Length Polymorphism (AFLPs) placed it as sister taxa of *S. longirostris* [21]. This conflicting evidence between different molecular and morphological characters has led to the present investigation. With the aim to understand the evolutionary mechanisms that may be behind the origin of *S. clymene*, namely a possible origin through natural hybridization, we sequenced one mitochondrial and six nuclear loci and followed a phylogenetics and population genetics approach. If *S. clymene* is of recent hybrid origin, we expect to find genetic intermediacy between the two parental forms, nuclear admixture, mitochondrial capture and unique mtDNA variation.

## Materials and Methods

### Ethics statement

Procedures for ensuring animal welfare during biopsy sampling were approved as part of the Scientific Research permits issued by the National Marine Fisheries Service under the authority of the Marine Mammal Protection Act of 1972 (16 U.S.C. 1361 et seq), the regulations governing the taking and importing of marine mammals (MMPA) (50 CPR part 216), the Endangered Species Act of 1973 (ESA) (16 U.S.C. 1531 et seq.), and the regulations governing endangered fish and wildlife permits (50 CFR parts 222–226). Biopsies were taken under NMFS permit numbers 14097, 774–1437, 774–1714, 1026/689424, and 873 issued to the National Marine Fisheries Southwest Fisheries Science Center. The samples originating from outside US jurisdiction were imported under CITES Import permit numbers US774223 and US689420, and under CITES Certificate of Scientific Exchange #690343. CITES permits are issued by the U.S. Fish & Wildlife Service. The Southwest Fisheries Science Center is a Registered Scientific Institution under CITES (US052).

### DNA extraction and sequencing

Tissue samples (skin or muscle) from *Stenella longirostris*, *Stenella coeruleoalba* and *Stenella clymene* were obtained from free-range dolphins using a dart biopsy system or from dead, stranded individuals. Fifty-eight samples were received as DNA extraction from the Southwest Fisheries Science Center, Marine Mammal and Turtle Research Sample Collection (SWFSC-NOAA, La Jolla, CA). DNA from the remaining samples was extracted following a standard proteinase K and two phenol-chloroform-isoamyl extractions [23]. Samples from *Stenella coeruleoalba* and *S. longirostris* used in this study were obtained from different locations in the Atlantic, Pacific and Indian oceans in order to provide an overall estimate of genetic diversity for each species. Samples from *Stenella clymene* were mostly obtained from the Gulf of Mexico and adjacent Atlantic Ocean waters and included a mass stranding that occurred near Tarpon Springs, in Florida (USA), in 1995.

For the mitochondrial DNA (mtDNA), a total of 72 individuals (*Stenella clymene*, n = 15; *S. longirostris*, n = 21; *S. coeruleoalba*, n = 36) were amplified and sequenced for the cytochrome *b* gene (1106 bp) using the L-strand primer on tRNA glutamine (L14724, 5′-TGACTTGAARAACCAYCGTTG 3′) and the H-strand primer on tRNA threonine (5′CCTTTTCCGGTTTACAAGAC 3′) [22] (GenBank Accession Numbers KF691950 - KF692018). Polymerase Chain Reactions (PCR) and sequencing conditions used are described in [24]. In addition, four anonymous nuclear loci [Del_05, Del_10, Del_12 and Del_16 [25]] and two introns [BTN [26] and PLP [27]] were PCR amplified and sequenced as described in [28] (GenBank Accession Numbers KF691817 – KF691949). Some samples failed to sequence for some of the loci. All PCR products were cleaned by adding 0.5 U of Shrimp Alkaline Phosphatase and 5 U of Exonuclease I and incubated at 37°C for 30 min and 80°C for 15 min. Both strands were directly sequenced (BigDye Terminator CycleSequencing; Applied Biosystems) on an ABI 3730 automated sequencer. All sequences obtained were aligned using the software Sequencher, version 5.0 (Gene Codes Corporation). An extended dataset, including additional samples from *Delphinus delphis, D. capensis*, *D. capensis tropicalis*, *Stenella attenuata, S. frontalis, S. longirostris, S. coeruleoalba*, *S. clymene, Tursiops truncatus, T. aduncus, Lagenodelphis hosei, Sousa chinensis*, *Sotalia fluviatilis*, *Globicephala melas* and *Phocoena phocoena*, sequenced for nine nuclear loci (the ones mentioned above plus CHRNA1, Del_04 and Del_11, see [13]) was used to estimate a species tree of the subfamily Delphininae using the method implemented in the software package *BEAST [29]. 1 000 million MCMC generations sampling every 100 000 generations were run in the program BEAST v. 1.7.4. [30], choosing the Yule process as the species tree prior, the Piecewise constant and linear model for population size estimates and a strict molecular clock with an uncorrelated lognormal distribution. The program Tracer v.1.5 was run to ensure mixing and that there was no lack of convergence of the posterior distribution and parameters by examining effective population size (ESS) values. TreeAnnotator v. 1.6.1. was subsequently used to summarize the obtained trees in a single tree that best represents the posterior distribution.

The same dataset sequenced for the cytochrome *b* gene was used to estimate a Bayesian phylogenetic tree using the program MrBayes v. 3.2.1 [31]. 5 million MCMC generation sampling every 1 000 generations were run. The dataset was partitioned by codon positions and the Tamura-Nei model of nucletodide substitution was chosen, according to the results given by Modeltest v. 3.8. [32]. Sequences from *Globicephala melas* and *Phocoena phocoena* were used as outgroups.

Heterozygous individuals found in the nuclear loci were resolved using the program PHASE v.2.1. [33], [34]. Alleles were inferred setting the phase-certainty threshold to 90%.

Nucleotide and haplotype diversities for each mitochondrial and nuclear locus, and for each species, were estimated in DNAsp v. 5.10.01 [35]. In order to assess the degree of genetic differentiation between the three *Stenella* species, pairwise *F*_ST_ was estimated for each locus with 10 000 random generations using Arlequin v. 3.5 [36]. Genealogical relationships at the haplotype level for each locus were inferred using the median-joining network as implemented in Network v. 4.6.0.0 [37].

In order to understand the phylogenetic relationships of the three species within the subfamily Delphininae, a species tree based on nine nuclear loci was estimated using a multi-locus species tree approach. A Bayesian phylogenetic tree was additionally estimated for the cytochrome *b* data

Morphological data were collected from 12 *S. clymene* individuals that were part of the mass stranding in Florida. Photographic data were available, which allowed the external appearance of the individuals to be compared and confirm the identity of two individuals that contain mitochondrial DNA of *S. longisrostris.* In addition, these dataset allowed us to verify if skull measurements correspond to the ones described in the species description of *Stenella clymene* [20]. Skull measurements were taken as described in [20].

## Results

In total, 72 sequences were obtained for the mitochondrial cytochrome *b* gene for the three *Stenella* species, which grouped into 61 haplotypes (Table 1). No haplotypes were shared among the three species. For the six nuclear loci, the number of sequences obtained for each species for each locus varied from 3 *S. clymene* sequences obtained for Del_05 to 17 *S. coeruleoalba* sequences obtained for BTN (Table 1). Some samples were not successfully phased into alleles and were therefore removed from subsequent analyses (Table 1). We shared haplotypes among the three species in two of the six nuclear genes, as also seen in the haplotype networks (see below).

**Table 1:** Number of *Stenella clymene, S. coeruleoalba* and *S. longirostris* specimens sequenced for this study.

	mtDNA	nuDNA					
	cytb	BTN	PLP	Del_05	Del_10	Del_12	Del_16
*S. clymene*	15	6/5	4/4	3/3	4/4	5/4	5/5
*S. coeruleoalba*	36	17/16	7/7	8/8	7/7	10/10	11/8
*S. longirostris*	21	11/10	10/9	8/7	6/6	9/9	11/11
Total samples	72	34/31	21/20	19/18	17/17	24/23	27/24
Total sites	1107	495	646	653	401	735	718
Variable sites	121	8	12	12	4	17	17

Numbers indicated after the slash correspond to the total number of samples used for analyses after removing samples that failed to be phased.

Levels of genetic diversity found in the mtDNA were high for all species, with *S. clymene* showing the highest nucleotide diversity, but the lowest haplotype diversity (Table 2). For the nuclear DNA, levels of genetic diversity obtained varied among loci, as expected given the stochasticity of the nuclear genome (Table 2). Nevertheless, levels found are within the range reported for nuclear loci obtained in other dolphin species (e.g. [28]).

**Table 2:** Levels of genetic diversity obtained for *Stenella clymene, S. coeruleoalba* and *S. longirostris* for the mitochondrial DNA (mtDNA) and for six nuclear loci.

a) mtDNA	π	Hd
Scly	0.01319+−0.00475	0.886+−0.069
Scoer	0.00872+−0.00134	0.967+−0.019
Slong	0.00884+−0.00104	0.995+−0.016
b) BTN		
Scly	0.00214+−0.00142	0.756+−0.130
Scoer	0.00247+−0.00030	0.806+−0.048
Slong	0.00250+−0.00028	0.805+−0.054
c) Del_05		
Scly	0.00155+−0.00047	0.800+−0.172
Scoer	0.00222+−0.00058	0.750+−0.107
Slong	0.00085+−0.0003	0.495+−0.151
d) Del_10		
Scly	0.00196+−0.00063	0.607+−0.164
Scoer	0.00101+−0.00043	0.385+−0.149
Slong	0.00138+−0.00016	0.545+−0.062
e) Del_12		
Scly	0.00569+−0.00095	0.964+−0.077
Scoer	0.00145+−0.00035	0.705+−0.111
Slong	0.00101+−0.00024	0.601+−0.113
f) Del_16		
Scly	0.00350+−0.00056	0.978+−0.054
Scoer	0.00186+−0.00030	0.850+−0.060
Slong	0.00239+−0.00034	0.874+−0.038
g) PLP		
Scly	0.00370+−0.00056	0.929+−0.084
Scoer	0.00167+−0.00032	0.758+−0.084
Slong	0.00197+−0.00042	0.732+−0.096

π nucleotide diversity; Hd – haplotype diversity.

The three species showed high levels of differentiation among them (Table 3). In the mtDNA, *S. clymene* and *S. coeruleoalba* were the least differentiated. In the nuclear DNA, levels of differentiation varied among loci, with *S. clymene* and *S. longirostris* being the least differentiated in 3 of the 6 loci (Table 3).

**Table 3:** Pairwise fixation index values (*F*_ST_) obtained between *Stenella clymene, S. coeruleoalba* and *S. longirostris* for the mitochondrial DNA (mtDNA) and for six nuclear loci.

a) mtDNA		
	Scly	Scoer
Scly		
Scoer	0.43892***	
Slong	0.71155***	0.80717***
b) BTN		
	Scly	Scoer
Scly		
Scoer	0.10393*	
Slong	0.01041	0.11836***
c) Del_05		
	Scly	Scoer
Scly		
Scoer	0.66941***	
Slong	−0.01483	0.56731***
		
d) Del_10		
	Scly	Scoer
Scly		
Scoer	0.38882**	
Slong	0.41950**	0.44734**
e) Del_12		
	Scly	Scoer
Scly		
Scoer	0.45211***	
Slong	0.488556***	0.44955***
f) Del_16		
	Scly	Scoer
Scly		
Scoer	0.53299***	
Slong	0.15699**	0.648***
g) PLP		
	Scly	Scoer
Scly		
Scoer	0.36231***	
Slong	0.33817***	0.15106**

*P*<0.01; ***P<0.001.

**Figure 1:**
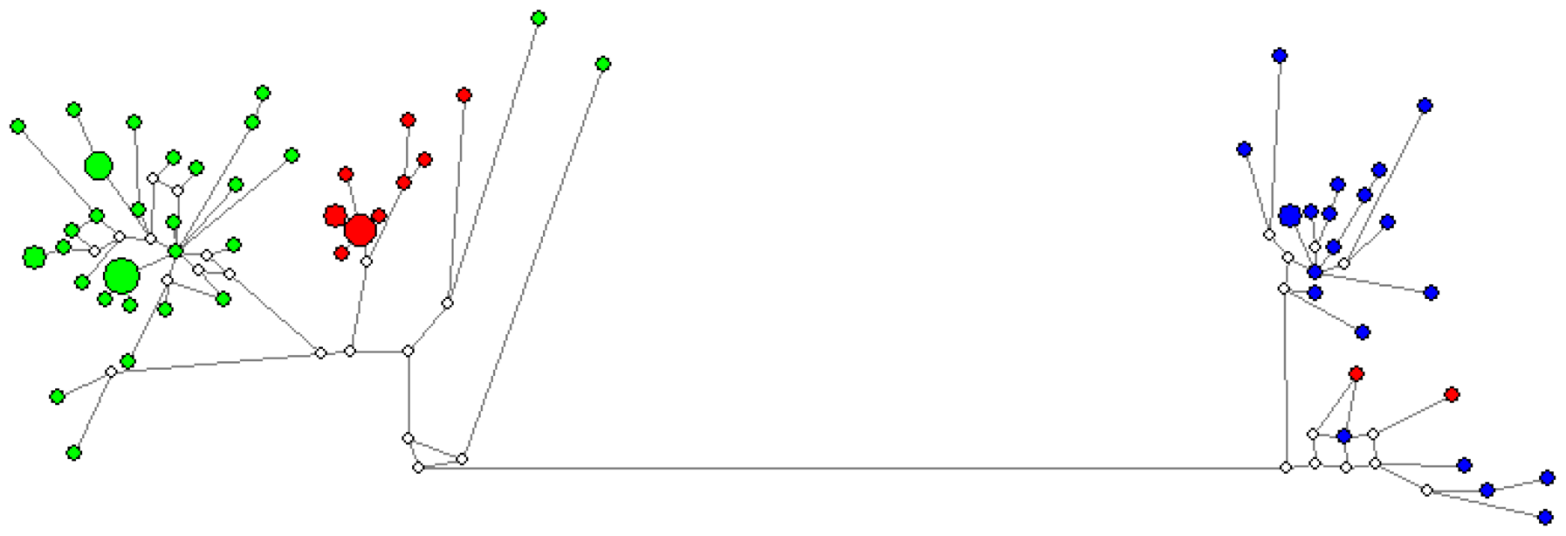
Median-joining network of cytochrome *b* haplotypes of *Stenella clymene* (red circles), *S. coeruleoalba* (green circles) and *S. longirostris* (blue circles). Circle size is proportional to the number of individuals exhibiting the correspondent haplotype. Length of lines separating the haplotypes is proportional to the number of mutational steps. White circles indicate missing, intermediate haplotypes.

**Figure 2:**
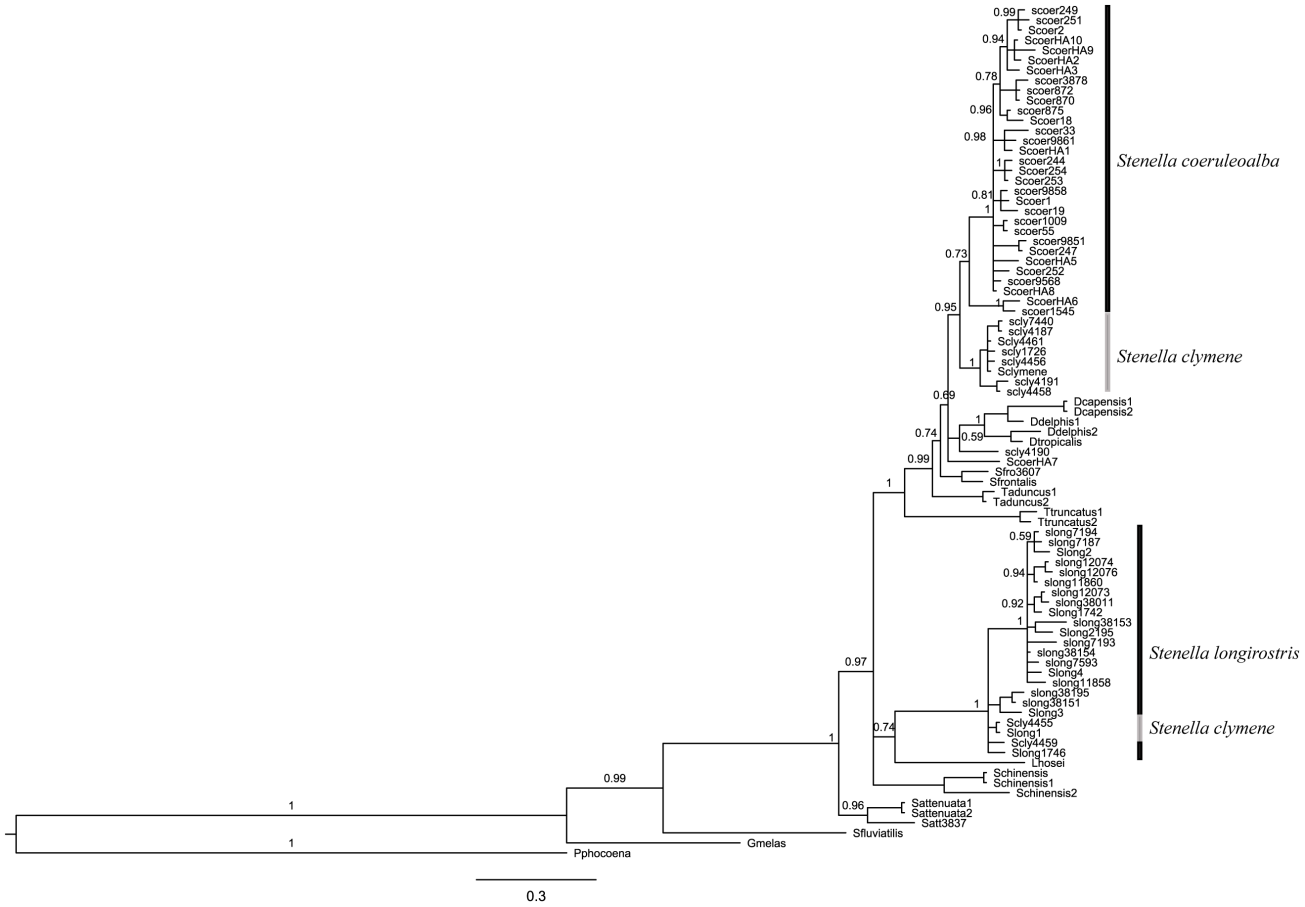
Bayesian phylogenetic tree generated in MrBayes for the cytochrome *b* gene. Posterior probability values are above nodes. Branch lengths are in substitutions/site.

The mtDNA haplotype network showed a clear separation between *S. coeruleoalba* and *S. longirostris*, with most *S. clymene* haplotypes being clustered among *S. coeruleoalba* (Figure 1). Two *S. clymene* haplotypes clustered with *S. longirostris*. The same pattern is seen in the mtDNA phylogenetic tree (Figure 2). In order to rule out misidentification or errors in amplification and sequencing of these individuals, DNA was re-extracted and amplification and sequencing was conducted in two different laboratories, and the same results were obtained.

**Figure 3:**
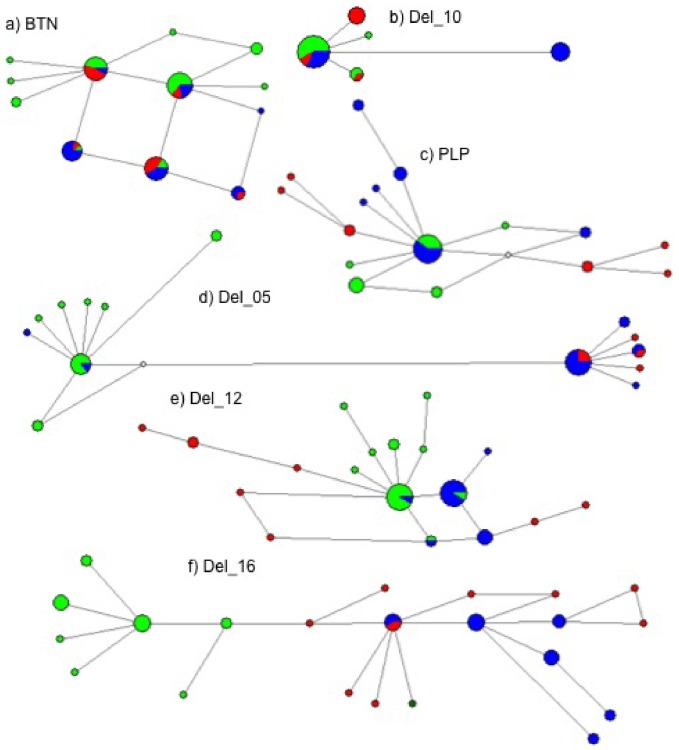
Median-joining networks of nuclear gene haplotypes of *Stenella clymene* (red circles), *S. coeruleoalba* (green circles) and *S. longirostris* (blue circles). a) BTN, b) Del_10, c) PLP, d) Del_05, e) Del_12, f) Del_16. Circle size is proportional to the number of individuals exhibiting the correspondent haplotype. Each species within each haplotype is coloured according to the legend. Length of lines separating the haplotypes is proportional to the number of mutational steps. White circles indicate missing, intermediate haplotypes.

Haplotype networks obtained for the six nuclear loci showed different patterns from the one obtained for the mtDNA (Figure 3). Two of the loci showed very little differentiation among the three species (BTN and Del_10), with shared haplotypes among all three. In contrast, the other four loci (PLP, Del_05, Del_12 and Del_16) showed a more clear separation, with fewer shared haplotypes. In PLP and Del_12, only *S. coeruleoalba* and *S. longirostris* share haplotypes, with *S. clymene* haplotypes occupying more external positions. In Del_05 and Del_16, *S. clymene* shares haplotypes with *S. longirostris*, clearly being more related to this species than to *S. coeruleoalba*. This pattern strongly contrasts from the one obtained in the mtDNA, where *S. clymeme* showed to be more related with *S. coeruleoalba* (Figure 1).

The two *S. clymene* individuals that clustered with *S. longirostris* in the mtDNA network share haplotypes with both *S. coeruleoalba* and *S. longirostris* in the least variable nuclear loci, but in the more informative loci are located in the tips of the networks, with the exception of Del_05 where they share a haplotype with *S. longirostris*.

**Figure 4:**
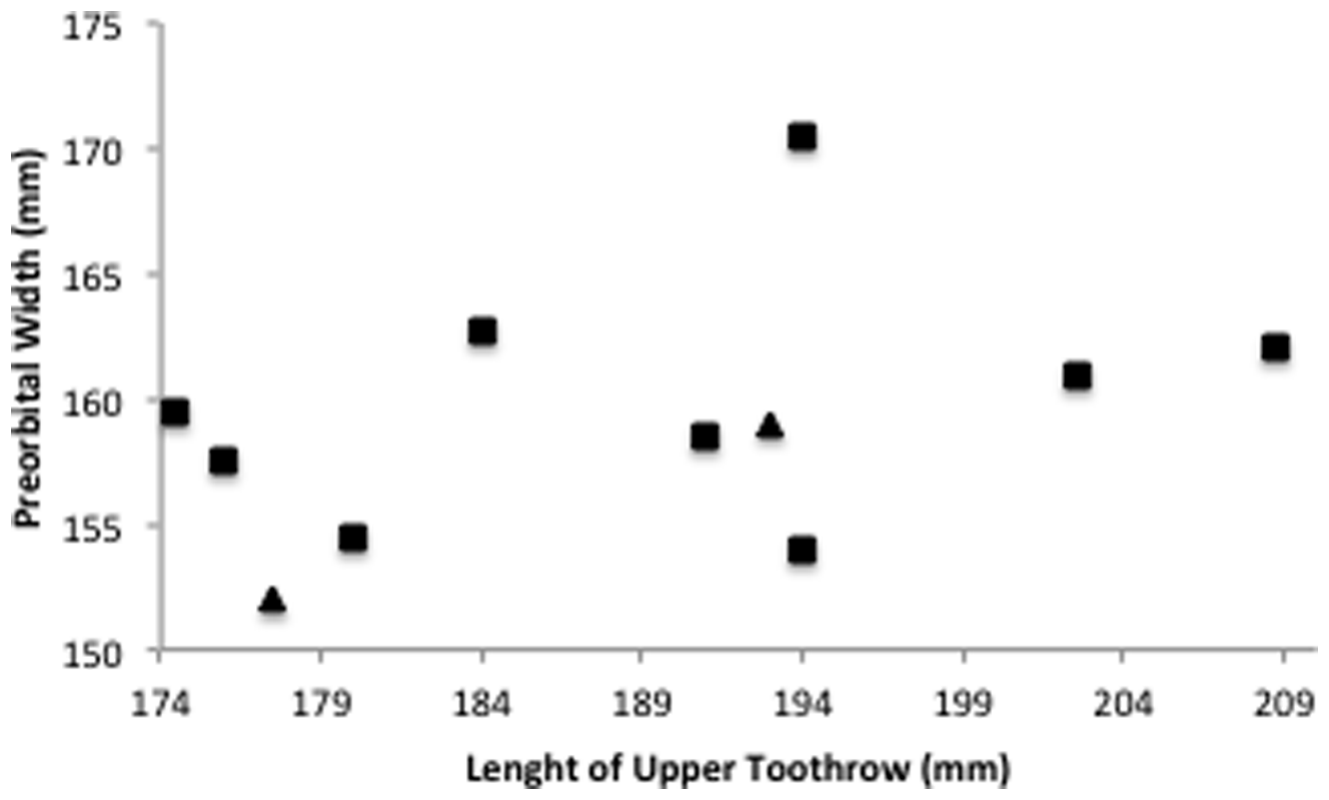
Scatterplot of preorbital width of the skull against length of the upper toothrow for 12 specimens of *Stenella clymene* stranded in Florida in 1995. Triangles represent the two hybrid individuals identified in this study.

These two individuals, along with others that were sequenced in this study, were part of a mass stranding near Tarpon Springs, Florida (USA) in June 1995. In order to confirm their morphological identification as *S. clymene*, we examined the available skulls, photographs of external appearance and biometric data collected by the Mote Marine Laboratory at the time of the stranding. Twelve skulls were measured in order to obtain the same characteristics described in [20] (Table S1), and a graph of the preorbital width against the length of the upper toothrow was plotted (Figure 4). This relationship summarizes differences in shape known to differentiate the three species [20]. The scatterplot shows that all individuals fit within the measurements described for *S. clymene*. Preorbital width varied between 152 and 170.5 mm (described range is 156–171 mm) and length of the upper toothrow varied between 174.5 and 208.75 mm (described range is 183–210 mm). Information on these twelve individuals is included in Table S1. The available photographs of the external appearance do not shed much light into the coloration patterns (Figure S1). Apart from the two putative hybrids, the other individuals that were part from the mass stranding had mitochondrial DNA of *Stenella clymene*.

**Figure 5:**
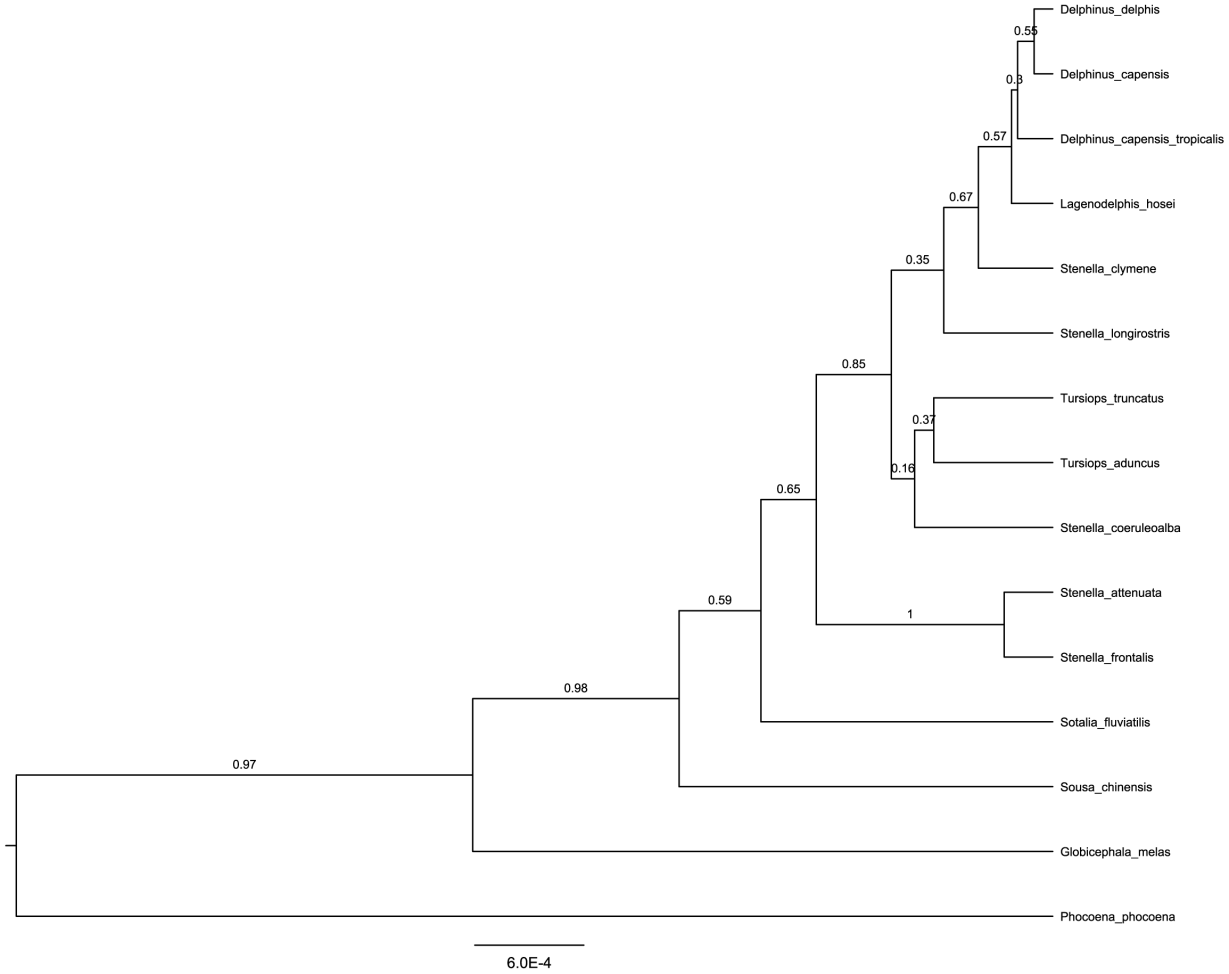
Species tree estimated with the *BEAST method. Posterior probability values are above nodes.

The species tree obtained with the nuclear loci resulted in a topology similar to the one recently published by [13], except for the branches including the genera *Delphinus*, *Lagenodelphis* and *Tursiops* and the three *Stenella* species analysed in this study (Figure 5). These branches had a low posterior probability value, reflecting an uncertainty of the method positioning the taxa. The study mentioned above did not include *Stenella clymene*, which may explain the differences observed in the tree here presented. Nevertheless, the results confirm that *S. clymene* is phylogenetically closer to *S. longirostris* than to *S. coeruleoalba*, although a relatively low posterior probability value (0.86) separates these two clades.

## Discussion

Our results suggest that the evolution of *Stenella clymene* does not follow a simple bifurcating tree, but more likely the result of reticulation through the admixture between two other closely related species, *S. coeuruleolba* and *S. longisrostris*. This finding has important implications not only for the clarification of the evolutionary relationships among species of the genus *Stenella* but also for our understanding of hybrid speciation in mammal species.

We confirmed that the mitochondrial genome of *Stenella clymene* is more closely related to that of *S. coeruleoalba* whereas the nuclear genome seems to be more closely related to *S. longirostris*. This result had already been described in previous phylogenetic studies, albeit with a smaller sample size and different molecular markers [21], [22]. This discrepancy of results between mitochondrial and nuclear markers suggests a hybrid origin of *S. clymene*, as a result of an ancient hybridization between a female *S. coeruleoalba* and a male *S. longirostris.* Our results further indicate that *S. clymene* is currently genetically distinct from its parental species, although backcrosses may still occur. We found two *S. clymene* haplotypes clustered with *S. longirostris* in the mitochondrial trees. These two individuals were part of a mass stranding of clymene dolphins in Florida. Their identification was confirmed by skull anatomy, external biometric measurements and coloration patterns, as explained in the Results section. The majority of the individuals have a mitochondrial DNA of *S. clymene*, except for the two individuals that appear to be the result of introgression between *S. clymene* and *S. longirostris*.

The morphological characteristics of *S. clymene* further support its origin through hybrid speciation. The morphometric variation seen in this species is outside that of both its putative parental species [20], suggesting a pattern of transgressive segregation [38], [39]. This pattern arises in later generation hybrids where parental alleles have recombined to form new genetic associations. These new combinations lead to novel phenotypes, which can sometimes explore new habitats and resources, contributing to niche divergence [40]. Although transgressive phenotypes can also be the result of selection following speciation, the most widely accepted phenomenon to originate evolutionary novelty is hybridization [41].

Ecological divergence has been suggested as one of the main drivers of hybrid speciation, leading to reproductive isolation between the hybrid and the parental species [5], [42]. This mechanism could explain the origin of *S. clymene*, although our knowledge on the ecology of this species is scarce and the few data available suggests that at least in some areas, the clymene dolphin may be exploring the same resources as *S. longirostris*. Mixed schools of both species have been observed in Florida and in the Caribbean [43]. Although ecological divergence has been suggested as critical for the persistence of a species of hybrid origin [5], [42], we suggest that behavioral mechanisms instead may be responsible for the maintenance of *S. clymene* as an independent lineage. Sexual selection, for example, can lead to assortative mating, resulting in a significant decrease in female mating with individuals from other species, and generating reproductive isolation [44]–[46]. This mechanism has been well demonstrated in primate, rodent and seal hybrid zones [11]. It is possible that mechanisms of female choice may have evolved in *S. clymene* as well. In this instance, *S. clymene* females would more often choose conspecific (hybrid) individuals rather than individuals from the parental species, even if encounters were frequent. Little is known about female choice in cetacean species, but this mechanism occurs among many other mammals. Baboon females, for example, have a preference for males that are phenotypically similar to themselves, regardless of ancestry [47]. Alternative mechanisms that have been proposed to explain the establishment of reproductive isolation between a hybrid and its parental species include chromosomal rearrangements and spatial isolation from the parental species. We do not consider these to be likely for *Stenella clymene*. Cetaceans, and dolphin species in particular, present strikingly similar karyotypes[15], which suggests a lack of major chromosomal rearrangement differences among the various species. The three *Stenella* species occur in sympatry, which also excludes the possibility of spatial isolation leading to reproductive isolation.

Several cases of hybrid speciation have been documented in animals [9], suggesting that this phenomenon may be more important than previously thought. Some of these cases have been found due to discrepancies in molecular markers, as the ones we found here (e.g. [45]). In mammals, only one case has been fully confirmed [38] but a handful of others suggested [48], [49], which shows that reticulate evolution is a viable mechanism for speciation in this group.

There are two alternative scenarios that could explain our results, although we consider them less likely: introgressive hybridization between *S. clymene* and *S. longirostris* such as the hybrid individuals that were part of the mass stranding in Florida, and non-reticulating forms of speciation including incomplete lineage sorting. If we had a case of introgressive hybridization, we would expect the hybrids to show intermediate morphological characters between the two species, which they do not. *S. clymene* has morphological characteristics of both *S. coeruleoalba* and *S. longirostris,* which lead to a novel phenotype. In addition, sporadic hybridization does not explain the strong phylogenetic discordance seen between mtDNA and nuDNA markers. Incomplete lineage sorting could explain the different patterns seen in the nuclear haplotype networks, and it is likely still affecting the nuclear genome of these species, given their recent divergence. However, it does not entirely explain the strong discordance between mtDNA and nuDNA markers or the morphological trait variation seen in *S. clymene*. Incomplete lineage sorting is usually found at any stage during a process of divergence or speciation, until populations or species are completely differentiated. However, even when this differentiation seems complete, we may still find portions of the genome where alleles are shared among populations or species, specially if there is ongoing gene flow (hybridization). Dolphin species within the subfamily Delphininae are known to have diverged quite recently, possibly during a rapid radiation event [13]. This coupled with a slow nuclear genome evolution which seems to be characteristic of cetacean species, leads to a general lack of polymorphism in nuclear loci and subsequent low levels of nuclear genetic differentiation among species [50].

The evolutionary relationships among species of the genus *Stenella* have been contentious to date, with morphological and nuDNA characters disagreeing with mtDNA [13], [22]. We suggest that this is likely due to the occurrence of natural hybridization among these species. This phenomenon is not easily incorporated into phylogenetic analyses, although some recent methods have been developed (e.g. [51], [52]), and until it is taken into account, a clear understanding of the relationships among these will not be possible.

Our study provides the first evidence of a marine mammal species that originated through hybridization between two other species. We suggest that sexual selection through assortative mating is likely the mechanism responsible for maintaining *Stenella clymene* as an independent lineage, despite ongoing backcrosses with the parental species. The permeability of the genome and the karyological uniformity of these species, coupled with a complex social structure and behavior that likely contribute to the establishment of reproductive isolation, suggest that hybrid speciation may be a more common evolutionary process in these species than previously thought. As more molecular studies become available, we expect to see additional cases of hybrid speciation reported in the near future. Such reports will provide evidence that, as with other animal complexes, reticulate evolution has been an important evolutionary process contributing to the diversity of marine mammals as well.

## Supporting Information

Figure S1 Photographs of external appearance of a *Stenella clymene* x *Stenella longirostris* hybrid (a, b) and of *Stenella clymene* individuals (c).
(TIF)

Table S1 Skull measurements for 12 *Stenella clymene* specimens stranded in Florida in 1995.
(DOCX)
