# Supplementary figures and images for: Hybrid Speciation in a Marine Mammal: The Clymene Dolphin (Stenella clymene)
Source: PLoS One. 2014 Jan 8;9(1):e83645. doi: 10.1371/journal.pone.0083645 (PMC3885441; doi:10.1371/journal.pone.0083645)

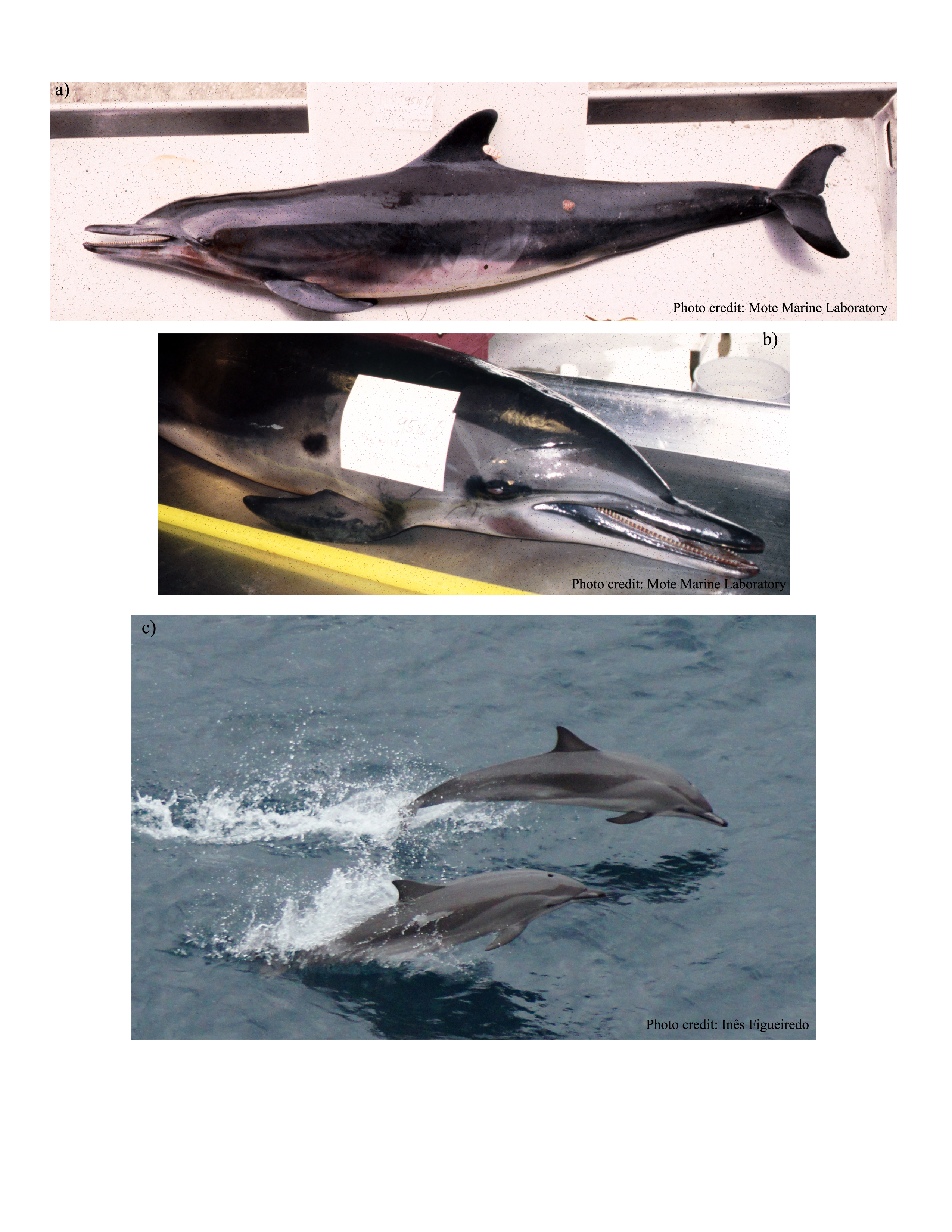

Supplement: Figure S1 — Photographs of external appearance of a Stenella clymene x Stenella longirostris hybrid (a, b) and of Stenella clymene individuals (c). (TIF) [file pone.0083645.s001.tif]
