# Supplementary material for: Hybrid Speciation in a Marine Mammal: The Clymene Dolphin (Stenella clymene)
Source: PLoS One. 2014 Jan 8;9(1):e83645. doi: 10.1371/journal.pone.0083645 (PMC3885441; doi:10.1371/journal.pone.0083645)
Supplement: Table S1 — Skull measurements for 12 Stenella clymene specimens stranded in Florida in 1995. (DOCX) [file pone.0083645.s002.docx]

**Supplementary Material**

Table S1. Skull measurements for 12 *Stenella clymene* specimens stranded in Florida in 1995.

| DNA code | 4185 | 4190 | 4191 | 4192 | 4193 |  | 4455 | 4456 | 4457 | 4459 | 4460 | 4530 |
| --- | --- | --- | --- | --- | --- | --- | --- | --- | --- | --- | --- | --- |
| Specimen code | MML9510A | MML9510G | MML9510H | MML9510I | MML9510J | MML9510K | MML9510L | MML9510M | MML9510N | MML9510O | MML9510P | MML9510Q |
| Sex | M | M | F | F | F | F | F | F | F | F | F | M |
| Total Lenght (cm) | 183 | 181.1 | 171 | 170.6 | 154 | 169.3 | 160 | 182.5 | 173 | 161 | 168 | 122 |
| Condylobasal length | 401(400) | 403(402.5) | 382 | 355 | 362(361) | 365(364) | 368(369) | 364 | 371 | 373(372) | 382(380) | 302(303) |
| Length of rostrum | 240(242) | 239(238) | 233(232) | 213(211) | 206 | 217(215) | 225(222) | 219(217) | 222(220) | 224(227) | 232(235) | 169(168) |
| Width of rostrum at base | 96(101) | 99 | 99(103.5) | 90(93) | 92(95.5) | 92(95) | 98(103) | 98 | 94(97) | 86(87) | 108(109) | 89 |
| Width of rostrum at 1/2 length | 51(50) | 54(56) | 56(55.5) | 51(53) | 53(54) | 55(58) | 50(48) | 53(56) | 52 | 49(48) | 52(54) | 57(55.5) |
| Width of pmx's at 1/2 length | 24 | 28 | 25 | 22(24) | 30 | 28(29) | 26(23) | 27(27.5) | 26(25.5) | 25 | 27 | 35 |
| Width of rostrum at 3/4 length | 41 | 46(48) | 42(41) | 40(43) | 46(45.5) | 44(47) | 41(38) | 43(48) | 43(44) | 40 | 43(44) |  |
| Greatest preorbital width | 162(160) | 163(161) | 171(170) | 158(157) | 160(159) | 155(154) | 152 | 163(162.5) | 160(157) | 150(148) | 154? | 138(136.5) |
| Greatest postorbital width | 177(174) | 182(181.5) | 188(189) | 169 | 178(177) | 176 | 172 | 176(174) | 175(175.5) | 169(165.5) | 184(182) | 146 |
| Greatest width of external nares | 37 | 44(45) | 41(42) | 41(39) | 39(40) | 35 | 39 | 44(43) | 39 | 40 | 45(46) | 37 |
| Zygomatic width | 173 | 179 | 186 | 168 | 177 | 176 | 172 | 171 | 172 | 165 | 180 | 143 |
| Greatest width of premaxillaries | 67(71) | 68(69) | 67(70) | 64(66) | 64(65) | 69(70) | 66(64) | 65(66) | 65(66) | 65(63) | 68(67) | 57(60) |
| Parietal width | 143 | 144(143) | 139(137) | 136(135) | 139(135) | 141(140) | 140(139) | 140(138.5) | 127 | 137(134.5) | 129(127) | 131(128) |
| Height of braincase | 109(112) | 11(108) | 101 | 99(101) | 103 | 106(103) | 99 | 94 | 99(94) | 92 | 99(100) | 90(94) |
| Internal length of braincase | 113 | 119 | 107 | 107 | 109 | 100 | 105 | 107 | 111 | 103 | 107 | 95 |
| Length of temporal fossa | (44 | (39 | (39 | (35 | (35 | (45 | (39 | (42 | (34 | (43 | (40 | (33 |
| Height of temporal fossa | (69 | (53 | (55 | (43 | (35 | (45 | (49 | (45 | (45 | (50 | (45 | (38 |
| Length of orbit | 44(48) | 48(50) | 46(48) | 40(43) | 47(49) | 46(48) | 46(47) | 46 | 43(49) | 46(48) | 49(52) | 40(43) |
| Length of preorbital process | 47(44) | 45(42) | 46(45) | 45(44) | 45(44) | 41(39) | 38(37) | 47 | 44(43) | 40(41) | 45 |  |
| Width of internal nares | 43 | 42 | 45 | 41 | 37 | 39 | 36 | 38 | 42 | 38 | 38 |  |
| Length of upper toothrow | 204(201) | 210(207.5) | 196(192) | 178(174) | 176(173) | 182(178) | 179(176) | 184 | 193(189) | 194(192) | 196(192) | 142(143) |
| Length of ramus | 339 | 347 | 333 | 304 | 319 | 316 | 322 | 313 |  | 320 | 342 | 253 |
| Height of ramus | 59(60) | 60(63) | 61(63) | 53(54.5) | 57(58.5) | 58(57.5) | 56(58) | 60(60.5) | 54(55.5) | 56 | 60(61) | 44 |
| Total no. of vertebrae | 72 | 72 | 74 | 71 | 70 | 71 | 73 | 71 | 73 | 73 | 71 | 72 |
| No. thoracic vertebrae | 13 | 13 | 13 | 13 | 13 | 14 | 13 | 13 | 13 | 13 | 13 | 13 |
| No. lumbar vertebrae | 20 | 21 | 25 | 23 | 21 | 19 | 22 | 22 | 23 | 22 | 21 | 22 |
| No. caudal vertebrae | 32 | 31 | 29 | 28 | 29 | 31 | 31 | 29 | 30 | 29 | 30 | 30 |
| Tooth counts: upper (average) | 37 | 36 | 38 | 38.5 | 35.5 |  | 37 | 36.5 | 38 | 36.5 | 34.5 | 42 |
| lower (average) | 39 | 40 | 41.5 | 40 | 38.5 |  | 40.5 | 39.5 | 38.5 | 40 | 36.5 | 43 |
